# Crucial Role of TSC-22 in Preventing the Proteasomal Degradation of p53 in Cervical Cancer

**DOI:** 10.1371/journal.pone.0042006

**Published:** 2012-08-01

**Authors:** Cheol-Hee Yoon, Seung Bae Rho, Seong-Tae Kim, Seongho Kho, Junsoo Park, Ik-Soon Jang, Seonock Woo, Sung Soon Kim, Je-Ho Lee, Seung-Hoon Lee

**Affiliations:** 1 Department of Life Science, Yongin University, Cheoin-gu, Yongin-si, Gyeonggi-do, Korea; 2 Division of AIDS, Center for Immunology and Pathology, National Institute of Health, Cheongwon-gun, Chungbuk, Korea; 3 Research Institute, National Cancer Center, Ilsandong-gu, Goyang-si Gyeonggi-do, Korea; 4 Division of Biological Sciences and Technology, Yonsei University, Wonju, Korea; 5 Korea Basic Science Institute, Daegeon Center, Daegion, Korea; 6 South Sea Environment Research Department, Korea Ocean Research and Development Institute, Geoje, Korea; 7 School of Medicine, Sungkyunkwan University, Samsung Medical Center, Seoul, Korea; The Chinese University of Hong Kong, Hong Kong

## Abstract

The p53 tumor suppressor function can be compromised in many tumors by the cellular antagonist HDM2 and human papillomavirus oncogene E6 that induce p53 degradation. Restoration of p53 activity has strong therapeutic potential. Here, we identified TSC-22 as a novel p53-interacting protein and show its novel function as a positive regulator of p53. We found that TSC-22 level was significantly down-regulated in cervical cancer tissues. Moreover, over-expression of TSC-22 was sufficient to inhibit cell proliferation, promote cellular apoptosis in cervical cancer cells and suppress growth of xenograft tumors in mice. Expression of also TSC-22 enhanced the protein level of p53 by protecting it from poly-ubiquitination. When bound to the motif between amino acids 100 and 200 of p53, TSC-22 inhibited the HDM2- and E6-mediated p53 poly-ubiquitination and degradation. Consequently, ectopic over-expression of TSC-22 activated the function of p53, followed by increased expression of p21^Waf1/Cip1^ and PUMA in human cervical cancer cell lines. Interestingly, TSC-22 did not affect the interaction between p53 and HDM2. Knock-down of TSC-22 by small interfering RNA clearly enhanced the poly-ubiquitination of p53, leading to the degradation of p53. These results suggest that TSC-22 acts as a tumor suppressor by safeguarding p53 from poly-ubiquitination mediated-degradation.

## Introduction

TGF-*β* stimulated clone 22 (TSC-22) was first identified as a TGF-*β*-inducible gene in mouse osteoblastic cells. TSC-22 expression is induced in a variety of cell lines by TGF-*β*, phorbol ester, serum, and progestin and positively regulates the TGF-*β* signaling [1], [2]. TSC-22 contains a leucine zipper-like motif, but it does not have a DNA-binding motif at the N-terminal region. TSC-22 can homodimerize and heterodimerize with TSC-22 homologous gene-1 (THG-1), and has transcriptional repressor activity [3].

Some researchers have identified the physiological roles of TSC-22 in the developmental process. TSC-22 is required for gastrulation during early embryogenesis in *Xenopus laevis* [4] and for oogenesis in *Drosophila* [5]. It has been also suggested that TSC-22 induces erythroid cell and Cardiac myofibroblast differentiation via activating the transcriptional activity of Smad3 and Smad4, and antagonizing the Smad7 in response to TGF-*β*-dependent signaling [6], [7].

Additionally, several studies have focused on the tumor suppression functions of TSC22. TSC22 is thought to be a potent tumor suppressor in salivary cancer cells [8], [9] human gastric carcinoma cells [10], hepatic carcinoma [11], human astrocytic tumors [12], and large granular lymphocyte leukemia [13]. The detailed mechanisms of the tumor suppressor function of TSC22 have been reported along with the hypothesis that TSC-22 represses the expression of the anti-apoptotic genes *Gadd45b* and *Lzts2* [11], negatively regulates Ras/Raf signaling [14] and involved in the TGF-b-mediated gastric carcinoma cell death in a caspase3-dependent manner [10]. On the other hand, TSC22-mediated apoptotic activity is inhibited by the interaction between TSC-22 and fortillin, followed by leading to TSC-22 destabilization [15].

**Figure 1:**
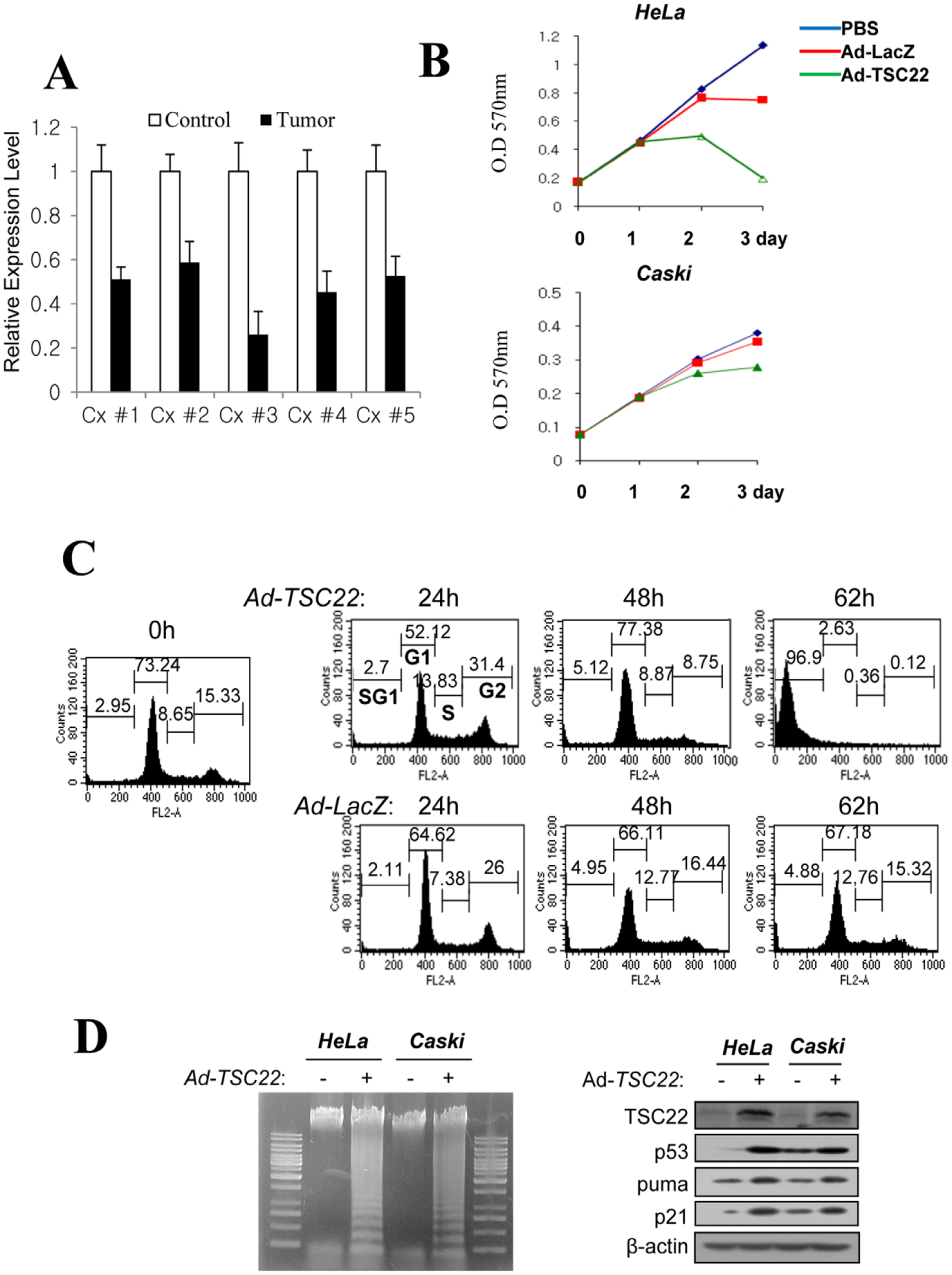
Expression of TSC-22 was decreased in human cancer tissue. (**A**) Total RNA was prepared from patients’ cancer specimens and the *TSC-22* mRNA level was then evaluated with real-time RT-PCR. Cx; serial number of patient tissue samples. (**B**) *HeLa* and *Caski* cells were plated on 6-well plates. After 24 h, the cells were infected with Ad-*TSC-22* or Ad-*LacZ*. At the indicated time points, cell numbers were determined by MTT assay to analyze cell proliferation rates. (**C**) *HeLa* cells infected with Ad-*TSC22* or Ad-*LacZ* were cultured for the indicated times, and the cells were then stained with propidium iodide (PI). The sub-G1 cell population (dead cell) and cell cycle profile of the PI-stained cells were analyzed by flow cytometry after PI-staining. (**D**) DNA fragmentation assay was performed by isolating chromosomal DNA from 1×10^6^ number of *HeLa* and *Caski* cells infected with Ad-*TSC22* or Ad-*lacZ* for 72 hrs (left). After 3 days infection of Ad-*TSC-22,* p53, Puma, p21, E6 and TSC22 expression were analyzed by Western hybridization (right).

p53 is a well-known tumor suppressor gene that acts by activating the transcription of its targeted genes such as p21, PUMA, , PKR and BAX [16]–[18]. p53 functions are regulated by post-translational modifications such as phosphorylation, acetylation, and ubiquitination. It is well-understood that p53 levels are tightly regulated by MDM2-mediated ubiquitination via an auto-regulatory feedback loop [19], [20]. Occasionally, the regulation of p53 expression is correlated with tumorigenesis through infection by human papilloma virus expressing E6, which leads to ubiquitin-mediated p53 degradation [21], [22]. Even though the regulation of p53 has been examined via multiple routes related to tumorigenesis, many questions remain about the tumor inhibition mechanism of the p53 pathway.

Since the mechanism underlying the network of tumor suppressor genes has not yet been explicitly elucidated, we conducted a cDNA microarray analysis of the gene expression profile in cancer tissue to find novel tumor-related genes. We found that TSC-22 expression was significantly decreased in cervical cancer tissues compared to normal tissues. Subsequently, we explored the novel function of TSC-22 during tumorigenesis. We therefore performed a yeast two-hybrid assay to screen for novel TSC-22-binding proteins. From this, p53 was identified as a TSC-22-binding protein. We also found that TSC-22 could enhance the activities of p53 through the inhibition of HDM2 and E6-mediated ubiquitination by directly binding to p53. On the other hand, TSC-22 over-expression stabilized p53 protein level, leading to increased cell death and inhibition of cell proliferation. Finally, tumor growth rate was strongly reduced by the expression of TSC-22 in a xenograft tumor model. Taken together, these results indicate that TSC-22 plays a crucial role in the inhibition of tumor growth through the regulation of p53 ubiquitination.

**Figure 2:**
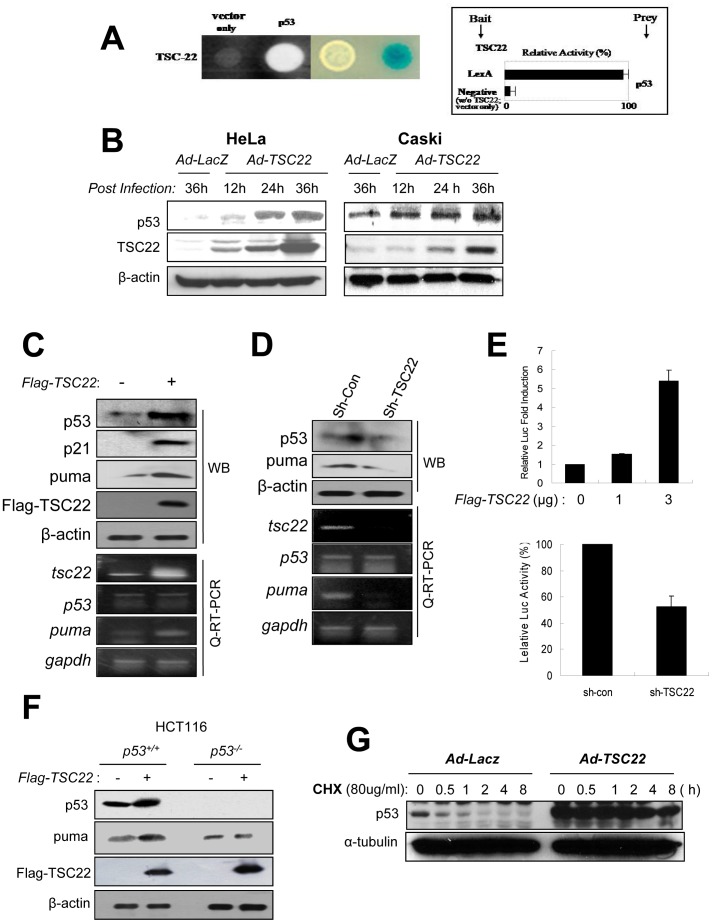
TSC-22 induces p53 expression. (**A**) TSC-22 and p53 cDNA constructs were cotransformed into EGY48 yeast cells to test for protein–protein interaction within the yeast two-hybrid system. Transformants were assayed for their ability to grow on medium lacking leucine (left) and for β-galactoside expression (right). (**B**) *HeLa* cells and *Caski* cells were infected with Ad-*TSC-22* for the indicated times. Protein levels were analyzed by Western blot with the DO-1 antibody against p53, the anti-TSC-22 antibody, and the anti-β-actin antibody as a loading control. (**C**) *HeLa* cells were transfected with 1 µg of Flag-TSC-22 expression vector. 24 h post-transfection, p53, Puma, p21, and TSC22 expression were analyzed by Western blotting and semi-quantitative RT-PCR with protein and total RNA obtained from each cell line. (**D**) *HeLa* cells stably expressing shRNA specific for TSC-22 and non-targeting control shRNA were analyzed to determine the protein and mRNA expression levels of p53 and Puma. (**E**) Luciferase activity of the p53RE (responsible element)-driven promoter were assessed with transfection of the indicated amount of Flag-TSC-22 plasmid in *HeLa* cells (upper panel). Activity of the p53RE-promoter was assessed in *sh-con* and *sh-TSC-22* expressing *HeLa* cells (lower panel) by luciferase assays. (**F**) One µg of Flag-TSC-22 or Flag-mock vector was transfected into *p53* ^+/+^ or *p53* ^−/−^ *HCT116* cells. At 48 h post-transfection, cell lysates were analyzed by Western blotting with the indicated antibodies. (**G**) Stability of p53 protein was assessed in *HeLa* cells infected with Ad-*TSC-22* or Ad-*LacZ*. 24 h after infection, cells were treated with cycloheximide (50 µg/mL) for the indicated periods of time. Cell lysates were analyzed by Western blotting with anti-p53 antibody (DO-1) with β-actin as a loading control.

## Results

### TSC22 Inhibits Tumor Growth

In order to analyze the specific gene expression profile of cervical cancer, we conducted cDNA microarray analysis with cDNA prepared from patients’ cervical cancer tissues. Interestingly, our microarray data showed that *TSC-22* gene expression was noticeably decreased in all cancer samples (data not shown). We then conducted real-time PCR (RT-PCR) analysis to confirm the microarray results. As shown in Figure 1A, *TSC-22* mRNA expression levels in the patients’ cancer tissues were significantly reduced compared to those in normal tissue.

These results led to further questions. The first question was whether TSC-22 could suppress tumor cell proliferation or not. Therefore, we infected *HeLa* (HPV-18) and *Caski* (HPV-16) cells with adenovirus expressing *TSC-22* or *LacZ* (infection control). As shown in Figure 1B, the proliferation ratios of both cells were significantly reduced by infection with Ad-*TSC-22*. We next examined whether TSC-22 could induce cell cycle arrest and apoptosis in *HeLa* cells. We found that the G0/G1 population was dramatically increased among Ad-*TSC-22-*infected cells compared to that in Ad-*LacZ-*infected cells within 48 h after infection. Furthermore, most Ad-*TSC-22-*infected cells underwent apoptosis in the late stage (Figure 1C). We next assessed the chromosomal-DNA fragmentation to observe the TSC-22-induced apoptosis in *HeLa* and *Caski* cells. As shown in Figure 1D, chromosomal DNA from Ad-*TSC-22*-infected *HeLa* and *Caski* cells showed very high level of fragmentation. Besides, p21 (cell cycle inhibitor) and PUMA (apoptosis inducer) expression levels were markedly enhanced in Ad-*TSC-22* infected *HeLa* and *Caski* cell (Figure 1D right panel). These effects were more significant in chronically HPV18-infected *HeLa* cell. p53 and TSC22 level in HeLa cells were barely detected compare(d) to caski cells (Figure 1D right panel). We speculate that their different expressions are caused by the different serotype of HPV. These results indicate that over-expression of TSC-22 induced high levels of cell death. Our results strongly suggest that TSC-22 plays a pivotal role in cervical tumor cell growth and death.

**Figure 3:**
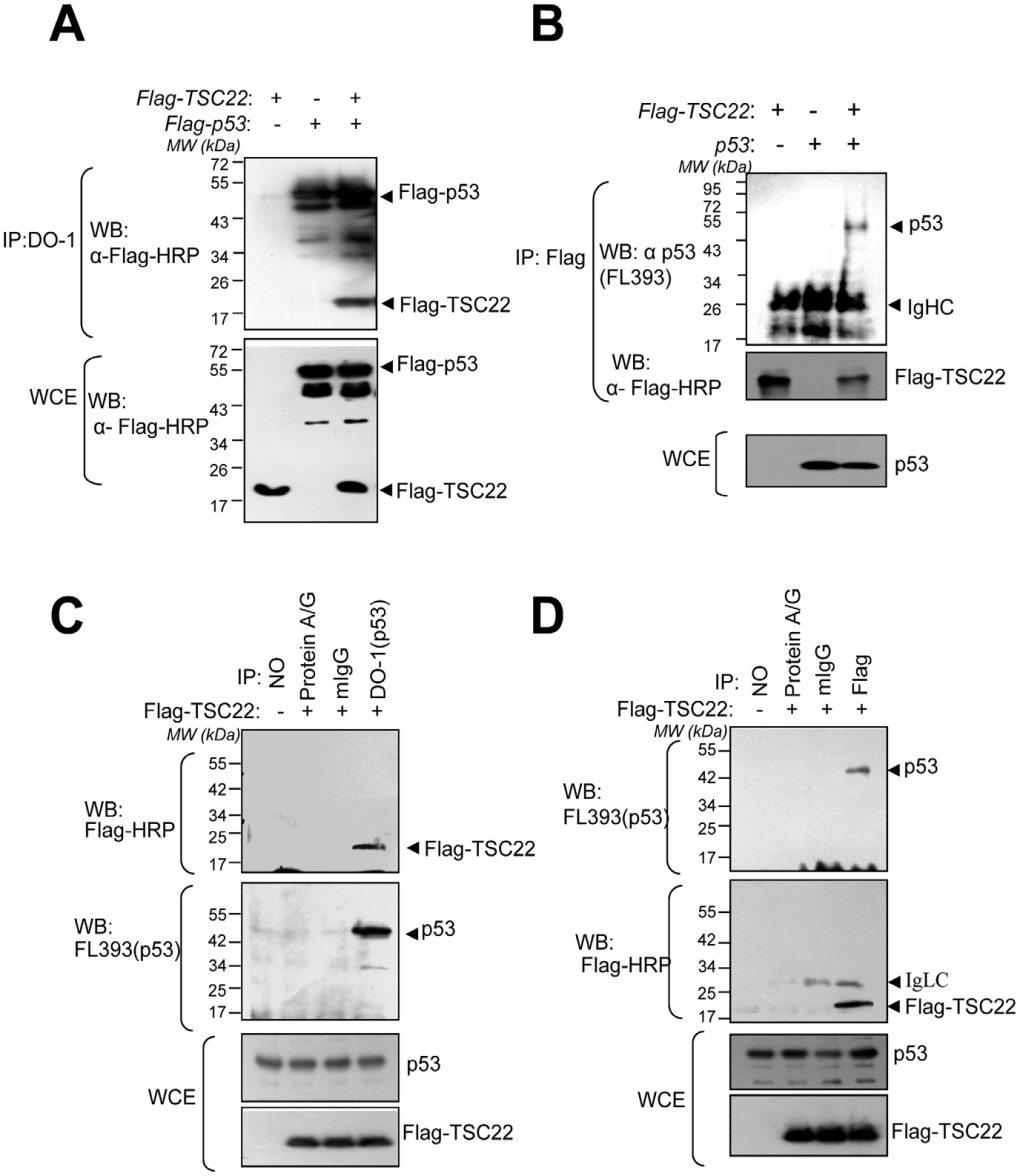
TSC22 interacts with p53 *in vivo.* (A–B) TSC22 interacts with ectopic p53. Ectopically expressed TSC-22 interacts with ectopically expressed p53 in *H1299* cells. Two µg of Flag-TSC-22 expression vector was cotransfected with Flag-p53 expression vector (A) or a non-tagged p53 expression vector (B) into *H1299* cells cultured in 100 mm plates. 48 h after transfection, cell lysates were immunoprecipitated with an anti-p53 (DO-1) (A) or anti-Flag (B) monoclonal antibody. Immunoprecipitates were analyzed by Western blotting using an HRP-conjugated anti-Flag (A) or anti-p53 (FL393) (B) monoclonal antibody. (**C–D**) **TSC-22 interacts with endogenous p53**. Ectopically expressed TSC-22 interacts with endogenous p53 in cells. HEK293 cells were transfected with 2 µg of Flag-TSC-22 plasmid for 48 h. Cell lysates were immunoprecipitated with anti-p53 (DO-1) monoclonal antibody, mouse immunoglobulin G (IgG) (C), or an anti-Flag (D) monoclonal antibody. Immunoprecipitates were analyzed with Western blotting using an HRP-conjugated anti-Flag (C) or anti-p53 (FL393) antibody.

### TSC-22 Binds to *p*53 in a Yeast Two-hybrid Assay

As shown by our data, TSC-22 contributes to the inhibition of cancer cell growth and cell death. This was consistent with previous studies demonstrating that TSC-22 is a potential tumor suppressor [8]–[11], [13], [23]–[25]. In order to elucidate the mechanisms underlying this function, we screened for TSC-22-binding proteins using a yeast two-hybrid assay. Through this, p53 was identified as a TSC-22-binding protein (Figure 2A, left panel). The interaction between TSC-22 and p53 was thus demonstrated *in vitro* by both cell growth and a β-galactosidase assay (Figure 2A, right panel).

### Determination of the Effects of TSC-22 on *p*53

To better understand the effects of TSC-22 on p53, *HeLa* and *Caski* cells were infected with Ad-*TSC-22* or *LacZ* for increasing periods of time. Interestingly, endogenous p53 levels were significantly increased by transfection of Ad-*TSC-22* in a time-dependent fashion (Figure 2B). The enhancement of p53 expression was more significant in *HeLa* cells than in *Caski* cells. In order to observe the enhancement of p53 target gene activity by the expression of TSC22, Flag-tagged TSC-22 was introduced into *HeLa* cells. p53 protein and its target genes, including p21 and puma, were clearly induced by Flag-*TSC-22* expression (Figure 2C). Conversely, knocking-down TSC-22 in *HeLa* cells reduced the protein levels of p53 and PUMA (Figure 2D). However, the level of p53 mRNA was not affected by knock-down and over-expression of TSC-22 (Figure 2C and 2D, bottom panel).

To determine whether p53 activity is regulated by TSC-22 expression in *HeLa* cells, we assessed the *p53RE* (responsible element)-driven promoter activity with TSC-22 expression and knock-down of TSC-22 in *HeLa* cells. A promoter harboring a *p53RE* was activated by TSC-22 expression in a dose-dependent manner. On the other hand, the promoter activity was decreased by TSC-22 knock-down. These data suggest that p53-mediated transcriptional activity is regulated by TSC-22 (Figure 2E). To evaluate whether decreases in PUMA and p21 are caused by direct regulation of p53 by TSC-22, Flag-tagged *TSC-22* plasmid was introduced into *HCT116 p53^+/+^* and *p53^−/−^* cells. Enhanced expression of PUMA was observed in *p53^+/+^* but not *p53^−/−^* cells (Figure 2F). This result suggests that the regulation of PUMA and p21 by TSC22 is dependent on p53. To further explore the enhancement of p53 by TSC-22, we assessed p53 stability in Ad-*TSC-22* or Ad-*LacZ* infected *HeLa* cells after cycloheximide treatment. As shown in Figure 2 G, Ad-*TSC-22* greatly enhanced and stabilized endogenous p53 levels. These results suggest that TSC-22 promotes the tumor suppressor function of p53 by enhancing the stability of p53 protein.

**Figure 4:**
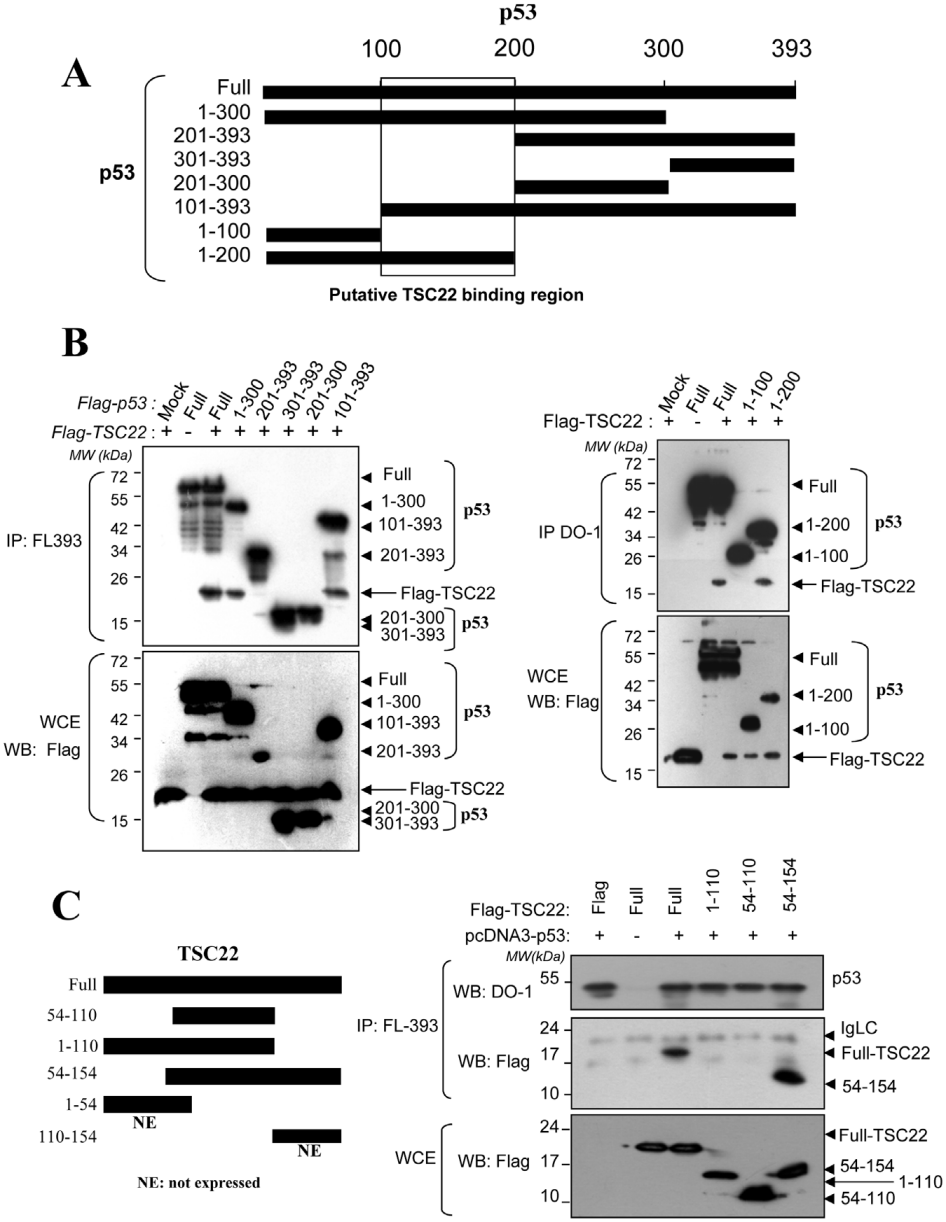
Mapping of the binding regions in TSC-22 and p53. (**A**) Schematic diagram shows the cDNA constructs for the Flag-tagged p53 deletion mutants and full-length p53, and indicates the TSC22 binding domain. (**B**) *H1299* cells were transfected with the indicated plasmids encoding Flag-tagged p53 deletion mutants along with the Flag-TSC-22 plasmid. Cell lysates were immunoprecipitated with the anti-p53 polyclonal antibody FL393 (left) or DO-1 monoclonal antibody specific for the N-terminal region (right), followed by Western blotting using the indicated antibodies. Lysates (5%) were also loaded onto an SDS gel for Western blotting using an anti-Flag antibody (bottom of each panel). (**C**) Schematic diagram showing the cDNA constructs of the Flag-tagged TSC-22 deletion mutants (left panel). *H1299* cells were transfected with the indicated plasmids encoding the Flag-tagged TSC22 deletion mutants together with Flag-p53 plasmid. Cell lysates were immunoprecipitated with an anti-p53 antibody (FL393); co-immunoprecipitated TSC22 was detected by Western blotting using the HRP-conjugated anti-Flag antibody (right, middle panel). Lysate (5%) was analyzed by Western blotting using an anti-Flag antibody (right, bottom panel).

### TSC-22 Binds to *p*53 *in vivo*

To determine whether TSC-22 interacts with p53 in mammalian cells, we transfected *H1299* human lung non-small cell carcinoma cells with the Flag-TSC22 expression plasmid and ^p53 expression plasmid, and then conducted co-immunoprecipitation and Western blot assays. We found that TSC-22 specifically co-immunoprecipitated with p53 in cells expressing both Flag-TSC22 and p53, but not in cells expressing either protein alone (Figure 3A). Conversely, p53 specifically co-immunoprecipitated with TSC-22 with the anti-Flag antibody (Figure 3B), suggesting an interaction between TSC-22 and p53. Next, we tried to confirm the endogenous interaction between p53 and TSC-22. Since we could not purchase an appropriate TSC-22 antibody to use in a co-immunoprecipitation experiment, this interaction was confirmed by reciprocal co-immunoprecipitation with endogenous p53 and exogenously expressed Flag-tagged TSC-22 in HEK293 cells which express high levels of p53 (Figures 3C and D). The results suggest that TSC-22 directly interacts with p53.

### TSC-22 Binds to p53 in the Region Including Amino Acids 100 to 200

To further define the region essential for binding TSC-22 to p53, we generated several p53 deletion mutants (Figure 4A). Expression plasmids of p53 and TSC-22 were transfected into *H1299* cells together or alone. As shown in Figures 4A and B, TSC-22 was able to bind to p53 several partial deletion mutants including p53^1–300^, p53^101–393^, and p53^1–200^. However, further deleting a portion of the internal region of the DNA binding domain, such as p53^1–100^, p53^201–393^, p53^301–393^ and p53^201–300^, abolished p53-TSC22 binding (Figures 4A and B). These results indicate that the region including amino acids 100–200, which is a part of the p53 DNA-binding domain, is required for binding TSC-22. Subsequently, to identify the p53 binding region of TSC-22, Flag-tagged truncated TSC-22 mutants that each contained an α-helix were expressed with p53 as shown in Fig. 4C. In co-immunoprecipitation experiments using an anti-p53 antibody (DO-1), TSC-22^54–154^ was co-immunoprecipitated with p53, but TSC-22^1–110^ and TSC-22^54–110^ were not. These data suggest that p53 binds amino acids 110 to 154 of TSC-22. Unfortunately, we could not further confirm the detailed interaction of p53-TSC-22 because TSC-22^110–154^ was not expressed in our experiment (Figure 4C). Taken together, our data demonstrate that TSC-22 and p53 interact at specific domains in each protein.

**Figure 5:**
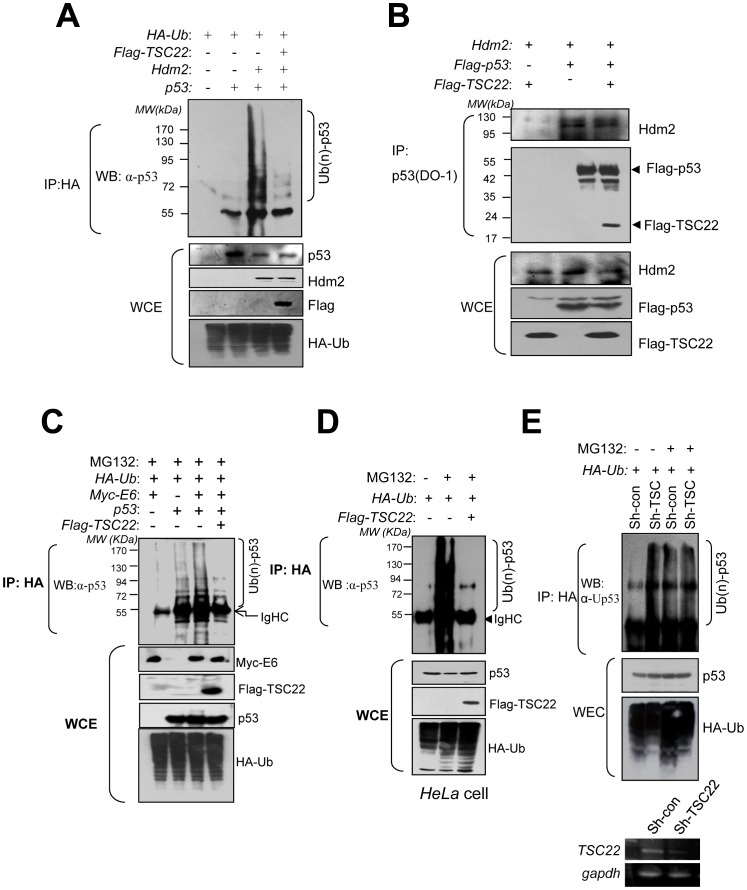
TSC22 inhibits HDM2- and E6-mediated p53 ubiquitination. (A) TSC22 inhibits HDM2-mediated p53 ubiquitination. *H1299* cells were transfected with the indicated plasmids. The transfected cells were treated with MG132 (20 µM) for 5 h before harvest. Cell lysates were immunoprecipitated with an anti-HA antibody. Ubiquitinated p53 was detected by Western blotting with an anti-p53 antibody (DO-1). Ubiquitinated p53 is indicated as Ub(n)-p53 (upper panel). The expression of total p53, HDM2, Flag-TSC-22 and HA-Ub proteins are shown in the lower panels. (B) TSC-22 does not interrupt interaction between p53 and HDM2. *H1299* cells were transfected with Flag-p53 along with Flag-TSC-22 or a Flag-mock vector in the presence of an HDM2 expression vector. Cell lysates were immunoprecipitated with the anti-p53 (DO-1) antibody followed by Western blotting using the indicated antibodies. Lysate (5%) was analyzed by Western blotting using indicated antibodies (lower panel). (C) TSC-22 inhibits E6-mediated p53 ubiquitination. *H1299* cells were transfected with the indicated plasmids in presence of an HA-Ub expression vector. 48 h after transfection, the transfected cells were treated with MG132 (20 µM) for 5 h before they were harvested. Cell lysates were immunoprecipitated with an anti-HA antibody. Ubiquitinated p53 was detected by Western blotting with the anti-p53 antibody (DO-1). Ubiquitinated p53 is indicated as Ub(n)-p53 (upper panel). The expression of total p53, Myc-E6, Flag-TSC-22, and HA-Ub proteins are shown in the lower panels. (D) TSC-22 disrupts ubiquitination of p53 in *HeLa* cells. *HeLa* cells were cotransfected with Flag-TSC-22 or a Flag-mock vector and HA-Ub expression vector. 48 h after transfection, cells were treated with 20 µM MG132 for 5 h prior to harvesting. Cell lysates were immunoprecipitated with an anti-HA antibody. Ubiquitinated p53 was detected by Western blotting with an anti-p53 antibody (DO-1). (**E**) Stable TSC-22 knock-down or control *HeLa* cells were treated with 20 µM MG132 for 5 h prior to harvesting. Ubiquitination of p53 was analyzed as described above.

### TSC22 Inhibits HDM2 and E6 -mediated p53 Poly-ubiquitination

To determine whether enhancement of p53 stability by TSC-22 is due to inhibition of p53 ubiquitination, *H1299* cells were transfected with HDM2, p53, and TSC-22 expression plasmids, and treated with the proteasomal inhibitor MG132 for 6 h in order to conduct *in vivo* ubiquitination assays**.** As shown in Figure 5A, p53 was highly ubiquitinated with the expression of HDM2. However, further HDM2-mediated p53 ubiquitination was significantly inhibited by the expression of TSC-22 (Figure 5A). We next wanted to determine whether the inhibition of HDM2-mediated p53 ubiquitination by TSC-22 is caused by interruption of the interaction between HDM2 and p53. Therefore, we introduced p53, HDM2, and TSC22 expression plasmids into *H1299* cells as shown in Figure 5B. p53 was then immunoprecipitated from the cell extracts with the DO-1 antibody. Interestingly, this experiment showed that p53 was simultaneously bound to HDM2 and TSC-22. In addition, the p53-HDM2 interaction was not interrupted by expression of TSC-22. These data suggest that TSC-22 can protect p53 from HDM2-mediated ubiquitination by directly binding to p53 in a region separate from the HDM2 binding site.

**Figure 6:**
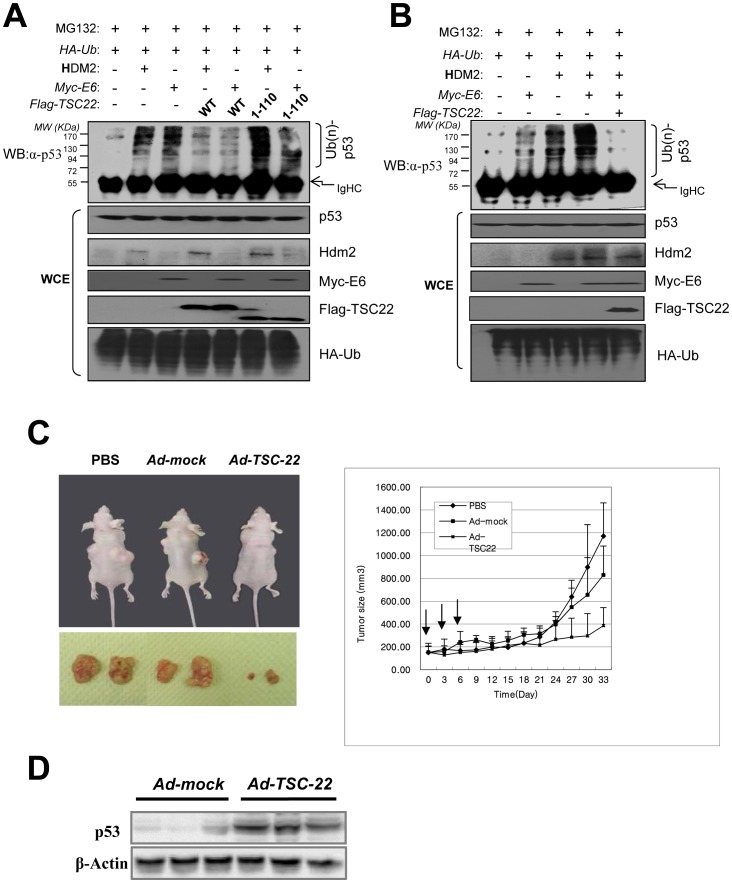
TSC-22 inhibits tumor growth in nude mice. (A) HDM2 and Myc-E6 were transfected with indicated plasmids into *H1299* cells. The transfected cells were treated with MG132 (20 µM) for 5 h before harvest. Cell lysates were immunoprecipitated with an anti-HA antibody. Ubiquitinated p53 was detected by Western blotting with an anti-p53 antibody (DO-1). Ubiquitinated p53 is indicated as Ub(n)-p53 (upper panel). The expression of total p53, HDM2, Flag-TSC-22 and HA-Ub proteins are shown in the lower panels. (B) Flag-tagged wild type (WT) or mutant TSC22^1–110^ was co-transfected with the indicated plasmids into *H1299* cells. The transfected cells were treated with MG132 (20 µM) for 5 h before harvest. Cell lysates were immunoprecipitated with an anti-HA antibody. Ubiquitinated p53 was detected by Western blotting with an anti-p53 antibody (DO-1). Ubiquitinated p53 is indicated as Ub(n)-p53 (upper panel). The expression of total p53, HDM2, Flag-TSC-22 and HA-Ub proteins are shown in the lower panels. (C) Nude mice were inoculated with 1×10^6^ *HeLa* cells by subcutaneous injection. Subcutaneous tumors derived from the *HeLa* cells were treated with adenovirus vectors as indicated. Tumor volumes are shown as the mean from at least five mice per group (n = 10 to 14 per group). Bars  =  SD. (D) Effect of TSC-22 treatment on the expression level of p53 in HeLa cell-derived tumors excised at the 27^th^ day post-treatment as determined by immunoblot analysis. Representative immunoblot of the three samples from each group.

We next attempted to show that TSC-22 can protect p53 from E6-mediated ubiquitination because the E6 and HDM2 binding domains of p53 overlap. p53 was highly ubiquitinated with the expression of E6 in *H1299* cells (Figure 5C, Lane 3); however, further expression of TSC-22 clearly blocked E6-mediated p53 ubiquitination (Figure 5C, Lane 4). We next used *HeLa* cells that constitutively expressed E6 to examine TSC-22-mediated p53 stability under physiological conditions. Ubiquitination of p53 was greatly increased after MG132 treatment in the cells. In contrast, this ubiquitination clearly disappeared upon over-expression of TSC-22 (Figure 5 D). p53 ubiquitination was rescued by the expression of shRNA specific for TSC-22 in *HeLa* cells in the absence of MG132 (Figure 5E, Lane 2). However, the difference in the level of p53 ubiquitination was not significant between *sh-con* and *sh-TSC-22* cells in the presence of MG132. We further explored the effect of TSC-22 on p53 ubiquitination by the expression of both HDM2 and E6. As shown in Figure 6A, the level of p53 ubiquitination was dramatically induced by both HDM2 and E6. However both of HDM2 and E6-mediated p53 ubiquitination was tightly interrupted by TSC-22 expression (Figure 6A). In addition, we tested whether TSC-22-p53 interaction is essential to inhibit p53 ubiquitination. As shown in Figure 6B, C-terminal deletion mutant TSC-22^110^ which does not interact with p53 (Figure 4C), did not inhibit the HDM2 and E6 mediated-p53 ubiquitination (Figure 6B). This result reveal that TSC-22 inhibit the p53 ubiquitination via direct interaction. These data strongly suggest that TSC-22 directly interacts with p53 and blocks the E6 and/or HDM2-medidated p53 ubiquitination, followed by stabilizing the protein of p53.

### TSC-22 Suppresses Tumor Growth in Nude Mice

We next determined whether TSC-22 inhibits tumor growth *in vivo*. Exponentially growing *HeLa* cervical cancer cells were injected subcutaneously into immune-deficient BALB/c nude mice. When the tumor volume reached about 100 mm^3^, 1×10^9^ pfu of adenovirus expressing TSC-22 or *LacZ* were injected into the tumors. After three sequential intra-tumoral injections of adenovirus, the animals (5–7 per group) were monitored for tumor growth. Tumor growth and morphology were analyzed over 30 days. Figure 6C shows that the tumor mass in mice injected with Ad-TSC-22 was remarkably reduced compared to tumors injected with Ad-*LacZ* or that were untreated. We next investigated the effect of TSC-22 on p53 stabilization in tumor tissues harvested from control and TSC-22 treated mice. TSC-22 transfection was observed to significantly increase the expression level of p53 protein (Figure 6D). Collectively, these results clearly demonstrate that TSC-22 can be a potent tumor suppressor in this animal model.

## Discussion

In our search to identify genes associated with cervical cancer development, expression pattern analyses following cDNA microarray experiments and RT-PCR revealed that the *TSC-22* gene was consistently reduced in tumor tissues (Fig. 1A). Several previous studies reported that TSC-22 is down-regulated in human salivary gland tumors [23], mouse liver tumors [11], and human brain tumors such as astrocytomas [26]. These results suggest that down-regulation of TSC-22 may play a role in the development of cancer in diverse tissues. However, these previous reports did not address the problem of TSC-22 being reduced in cancer cells. A reasonable explanation is that TSC-22 may inhibit cancer cell development. Our results showed that TSC-22 dramatically inhibited cell proliferation and induced cell death when expressed with an adenovirus expression system (Figures 1C and D). Similarly, enhancement of TSC-22 expression is associated with increased apoptosis in human gastric carcinoma cells [10].

Given that TSC-22 is a transcription repressor [3], the role of TSC-22 in tumor suppression has been suggested by Mari *et al*. [11]. Through experiments using TSC-22 siRNA, this group demonstrated that the DNA damage-inducible gene 45 β (Gadd45ß) and putative tumor suppressor 2 (Lzts2) are putative targets of TSC-22. However, the relationships through which these targets play pivotal roles in tumor suppression by TSC-22 were not directly revealed. In our attempt to find a novel function of TSC-22 in tumor suppression, we identified a binding protein in a yeast two-hybrid experiment. This study showed that TSC-22 directly binds to p53 (Figure 2A). We further observed that TSC-22 up-regulated the protein levels of p53 without altering the levels of p53 mRNA. TSC-22 over-expression and knock-down experiments showed that TSC-22 facilitates the function of p53 as a transcriptional activator of target genes that can inhibit tumorigenesis (Figure 2C–G). These results indicated that increased p53 protein levels are associated with post-translational regulation by TSC-22. TSC22-p53 interaction was assessed by *in vivo* assays in which p53 was co-immunoprecipitated with TSC-22 (Figure 3–4).

Over-expression of TSC-22 clearly prevented ubiquitination of p53 by HDM2, which regulates p53 turnover through its E3 activity. Several regulators that influence p53 function via modulation of the HDM2–p53 interaction have been identified, including p14Arf [27], [28], YY1 [29], gankyrin [30], L11 [31], Daxx [32], Numb [33], and Nucleostemin [34]. Among these regulators, Numb is most similar to TSC-22 because Numb binds to p53 and HDM2, thereby preventing ubiquitination and degradation of p53 [33].

Indeed, we detected a ternary complex that contains all three proteins: TSC-22, HDM2 and p53. Thus, TSC-22 does not compete with HDM2 for binding to p53. TSC-22 binds to the DNA binding domain of p53, which is essential for p53 function. Therefore, it remains to be determined whether this interaction is required to activate the target genes of p53 in the nucleus.

Interestingly, increased cell death and inhibition of proliferation by TSC-22 expression were more frequently observed in *HeLa* cells, which constitutively express E6, than in *Caski* cells which do not express E6 (Figures 1C and D). This seemed consistent with results shown in Figure 2B demonstrating that p53 levels were more significantly restored in *HeLa* cells by expression of TSC-22 than in *Caski* cells (Figure 2B).

Based on the E6-p53 interaction [21], p53 ubiquitination was spontaneously observed in *HeLa* cells even though the cells were not transfected with HDM2. However, p53 ubiquitination clearly disappeared upon over-expression of TSC-22 in *HeLa* cells (Figure 5C). In contrast, depletion of TSC-22 by shRNA induced p53 ubiquitination (Figure 5D).

We also performed quantitative RT-PCT and found the suppressed mRNA level of p53 and TSC-22 and high expression level of E6 mRNA in cervical cancer cell lines and patients’ tissue samples, but we could not find the physical correlation among them (Fig. S1). Although our results could not show the correlation of HPV/E6 and p53 protein expression level in cervical cancer tissues expressing less amount of TSC-22, it is well known that most of cervical cancer is caused by E6 expression after infection of human papillomavirus (HPV), followed by down-regulation of p53 [22]. Taken together, our results imply that TSC-22 can suppress the oncogenic potential of HPV by preventing the degradation of p53 by E6-mediated ubiquitination. However, how TSC-22 inhibits ubiquitination and degradation promoted by HDM2 and E6 remains to be investigated in the future. In an attempt to address the effect of TSC-22 on tumor suppression *in vivo*, we found that the growth of *HeLa* cells inoculated into nude mice was significantly reduced by TSC-22 adenoviral transfection (Figure 6C). This result suggests that TSC-22 could be targeted of future cancer gene therapies.

In conclusion, our study identified TSC-22 as a novel factor interacting with p53. We also showed that TSC-22 prevents the degradation of p53 protein by HDM2 and E6, which suggests a novel mechanism by which TSC-22 regulates apoptosis and cell proliferation. Our results also revealed a previously unrecognized mechanism underlying the effects of TSC-22 on tumor suppression, and demonstrated that TSC-22 is a possible new target for human cancer therapy.

## Materials and Methods

### Patient’s Samples

Cervical cancer tissues were obtained from patients of the Department of obstetrics and Gynecology, Samsung Medical Center, Seoul, Korea. All tissue samples were prepared during surgery and stored at −70°C. Before RNA extraction, a part of each tissue sample was sliced by paraffin section and examined by hematoxylin and eosin staining. Tissue samples containing more than 50% tumor cells were selected.

### Cell Lines and Mice

Immune-deficient BALB/c nude mice were purchased from Orient Bio (Gyeonggi, Republic of Korea). Human *293, H1299, HeLa, Caski, HT3*, *HCT116 p53* ^+/+^ and *HCT116 p53* ^−/−^ cell were cultured in recommended medium supplemented with 10% (V/V) fetal bovine serum (FBS), TSC22 knock-down *HeLa* cell was established by previous established method [18], [35]. In brief, TSC-*shRNA*-expressing recombinant plasmid (*sh-TSC*) was constructed by manipulation of the pSilencer3.1 vector (Ambion) with the ds-oligonucleotide prepared by annealing the following synthetic oligonucleotides: forward; GATCCGTGGATCTAGGAGTTTACCATTCAAGAGA TGGTAAACTCCTAGATCCATCTTTTTTGGAAA-3′ and reverse; 5′-AGCTTTTCCAA AAAAGATGGATCTAGGAGTTTACCATCTCTTGAATGGTAAACTCCTAGATCCACG-3′. After transfection of recombinant plasmid into *HeLa* cell, TSC22 knock-down *HeLa* cells were isolated by culture supplemented with 1 ug/ml of puromycin. DNA transfections were carried out using lipofectamine2000 (invitrogene), or Fugene HD (Roche), each according to the manufacturer’s instructions.

### Real-time and Semi-quantitative RT-PCR Analysis

Total RNA were obtained by extracting tissues in TRIzol reagent (Invitrogen) according to the manufacturer’s instructions. First strand cDNA was prepared from total RNA and oligo dT using I Script cDNA synthesis kit (Bio-Rad, USA). Real-time PCR was conducted with a Mini Opticon System (Bio-Rad) and SYBR Green (Bio-Rad). Specific primers used for Real-time PCR were 5′-GTAGACCAGTGGCGATGGAT and 3′-GCTACCACACTTGCACCAGA.

Semi-quantitative RT-PCR was performed with ONE-STEP^R^ RT-PCR kit (Intron Bioscience, South Korea). Specific primers used for quantitative RT-PCR were: human TSC22; forward 5′-GCTGCCGTTTTCTGTTTCTC-3′ reverse 5′-ATCCATCGCCACTGGTCTAC-3′, human p53; forward AAGTCCAAAAAGGGTCAG TCT reverse CCCAAACATCCCTCACAGTAA, human PUMA; forward 5′-GGAGGGTCCTGTACAATCTC-3′ reverse 5′-GCTACATGGTGCAGAGAAAG-3′: HPV-18 E6; forward 5′-ATGCTGCATGCCATAAATGT-3′ reverse 5′-TGCCCAGCTATGTTGTGAAA-3′, HPV-16 E6; forward 5′-TTGCTTTTCGGGATTTATGC-3′ reverse 5′-TCAGGACACAGT GGCTTTTG-3′, GAPDH; forward 5′- GACATCAAGAAGGTGGTGAA-3′, reverse 5′-TGTCATACCAGGAAATGAGC - 3′. Real-Time quantitative RT-PCR data was normalized to GAPDH mRNA, and analyzed by fold induction in comparison with a standard control for each experiment, and are represented as mean ± SEM of three independent experiments. Semi-quantitative RT-PCR data was shown by electrophoresis in agarose gel.

### Ethics Statement

All animal studies were approved by the Animal Care and Use Committee of Samsung Medical Center.

### Luciferase Assay

Luciferase assay were performed as described previously with minor modification [18]. Briefly, different amount of *Flag-TSC22* plasmid was co-transfected with 200 ng of p53RE-conjugated luciferase reporter plasmid (pGL-3, Promega) together with control *pCMV-lacZ* plasmid into the *HeLa* cells for 48 h. The luciferase activity was measured and normalized (to β-galactosidase) using Bright-Glo Luciferase Assay system (Promega) and the Genios Luminometer (TECAN, Austria). Data are represented by error bar ± SEM.

### Adenovirus and Vector Construction

PCR-amplified, full-length human TSC22 fragment (500 bp) was cloned into pCRII-TOPO vector. The primers used to generate the full-length were 5′-GGGTGTTTTTGGCTGCAAT and 3′-TTCAGTTCACACGCAGCAG. The cloning product was confirmed for sequence from both directions. The pCR-TOPO-TSC22 was digested with EcoRI and cloned into the pΔACMVp(A) EcoRI site. The pΔACMV-TSC22 and adenovirus backbone vector, pJM17 were cotransfected into a packaging cell line, 293 using Fugene HD transfection reagent (Boehringer Mannheim). A replication competent virus (RCV) negative clone was propagated in 293 cells and purified through two rounds of CsCl density gradient centrifugation. The Ad-TSC22 construct was used at 100 M.O.I. for all transfection experiments. AdCMV-*lacZ* was used as an internal control. Full and partial length cDNAs of human TSC22 and p53 were introduced into pcDNA3-Flag or pcDNA3 (Invitrogen), respectively. Human papillomavirus E6 (type 16) were cloned into pcDNA3-HA vector. pcDNA3-HDM2 vector and HA-Ubiquitin expression plasmids were pleasant gifted from J.W.Song (SKKU). DNA transfections were carried out using lipofectamine2000 (Invitrogen), or Fugene HD (Roche), each according to the manufacturer’s instructions.

### Cell Proliferation Assay

Cells were plated in triplicate at a density of 2×10^5^ cells/well in 6-well plates. Twenty-four hours later, the cells were infected with *Ad-TSC22* or *Ad-LacZ*. Beginning 24 h after infection, cells were counted each day by MTT assay for up to 3 days.

### Flow Cytometry and Apoptosis Assay

Flow cytometry analysis was carried out as described previously [18], [36]. In brief, cells infected with Ad-LacZ and Ad-TSC22 were harvested at indicated time point, fixed in 70% ethanol for 1 hr, and then stained with a propidium iodide (PI) solution containing RNaseA (Sigma) for 30 min at room temperature in the dark. Samples were then analyzed by FACS Calibur (BD Bioscience) with CellQuest software.

*HeLa* and *Caski* cells were plated onto six-chamber slides and infected with the indicated Ad-*LacZ* or Ad-*TSC22*. For the observation of nuclear apoptotic body, the nuclei were fixed in methanol and stained with 40, 60-diamidino-2-phenylindole (DAPI, Sigma-aldrich) for 15 min and rinsed twice with PBS, then examined with the fluorescence microscope.

### Western Blot and Antibodies

Samples were separated on 10% polyacrylamide-SDS gels. The gel was transferred overnight to nitrocellulose membrane and blocked in TBS-Tween-20 (0.1% v/v) with dry skimmed milk (5% w/v) (PBSTM) for 1 h at RT. And then western blotting was carried out by using respective antibody and ECL (Amersham). Antibodies against the following proteins were purchased; p53 mAb (DO-1), p53 pAb (FL393), HDM2 pAb, HA pAb (H9), HRP-conjugated Myc Ab, mouse IgG and rabbit IgG were obtained from Santa Cruz Biotechnology. p21waf/cip mAb (DCS60), PUMA pAb, was purchased from Cell signaling technology. Flag (or HRP conjugated) mAb (M2) and β-actin mAb were purchased from Sigma–Aldrich.

### *In vivo* Ubiquitination Assay

*In vivo* ubiqutination assay were performed as described [36]. In brif, *H1299* cells were transfected with plasmids as described in the legend to figure. The cells were treated with 20 µM of MG132 for 5 h and then harvested at 48 h post-transfection. Ubiquitinated proteins were immunoprecipitated by HA poly clonal antibody. And then ubiquitination of p53 was detected by western blotting by using p53 Antibody (DO-1).

### Yeast-two Hybrid Assay

LexA-human TSC22 fusion protein was constructed and used to screen binding proteins from a human ovary cDNA library (Clontech, Palo Alto, CA). The binding proteins were expressed as pB42 fusion proteins. cDNA encoding full length human p53 were PCR amplified and cloned separately into the EcoRI/XhoI sites of the B42. Experiment of yeast two hybrid assay was previously described [37]. In brief, positive interactions were confirmed by cell growth on leucine-depleted yeast synthetic medium and blue colony formation on 5-bromo-4-chloro-3-indolyl-h-D-galactoside (X-gal, 5 mmol/L)-containing medium. The activity of the interaction between TSC22 and p53 was determined by measuring the relative expression level of β-galactosidase. The β-galactosidase activity was calculated using the formula units  = [1,000×(A_420_−1.75×A_550_)]/(time×volume×A_600_).

### Co-immunoprecipitation

To perform co-immunoprecipitation *in vivo*, *H1299* cell were transiently transfected with expression plasmids of Flag-tagged TSC22 and Flag-tagged (or non-tagged) p53 or HDM2 if needed using lipofectamin 2000 reagent (invitrogen). The p53 was immunoprecipitated with anti-p53 antibody (DO-1 or FL393) and coimmunoprecipitated TSC22 or HDM2 were detected by Western blot with anti-Flag antibody (for TSC22) and HDM2 antibody respectively. For semi endogenous coimmunoprecipitation with p53 and TSC22, *HEK293* cells were transiently transfected with Flag-tagged TSC22 using lipofectamin 2000 reagent. Immunoprecipitation of p53 and Flag-TSC22 were carried out with anti-p53 (DO-1 or FL393) and Flag antibody, respectively. The co-immunoprecipitated Flag-TSC22 and p53 was detected by Western hybridization.

### S.C. Tumor Models

To establish tumors in mice, 1×10^6^ *HeLa* cells were injected s.c. in the mid-dorsal region of nude mouse. An intra-tumoral injection of 1×10^9^ pfu/40uL of Ad-TSC22 was done thrice. Tumor size was evaluated by caliper measurements every 3 days. Mice were sacrificed on day 27 after final virus injection. Tumors were then excised.

## Supporting Information

Figure S1 **Quantification analysis of p53, E6 and TSC-22 genes.** The mRNA expression of p53, E6 and TSC-22 in cervical cancer cell lines (A) and patients’ cancer specimens (B) was performed by quantitative RT- PCR by using GAPDH protein as reference gene.
(TIF)

## References

[1] Shibanuma M, Kuroki T, Nose K (1992) Isolation of a gene encoding a putative leucine zipper structure that is induced by transforming growth factor beta 1 and other growth factors. J Biol Chem 267: 10219–10224. 1587811

[2] Ahn H, Han T (2011) Regulation of TGF-beta signaling by PKC depends on Tsc-22 inducibility. Mol Cell Biochem *online published*. 10.1007/s11010-011-1042-8 21881999

[3] Kester HA, Blanchetot C, den Hertog J, van der Saag PT, van der Burg B (1999) Transforming growth factor-beta-stimulated clone-22 is a member of a family of leucine zipper proteins that can homo- and heterodimerize and has transcriptional repressor activity. J Biol Chem 274: 27439–27447. 10488076 10.1074/jbc.274.39.27439

[4] Hashiguchi A, Okabayashi K, Asashima M (2004) Role of TSC-22 during early embryogenesis in Xenopus laevis. Dev Growth Differ 46: 535–544. 15610143 10.1111/j.1440-169x.2004.00770.x

[5] Dobens LL, Hsu T, Twombly V, Gelbart WM, Raftery LA, et al (1997) The Drosophila bunched gene is a homologue of the growth factor stimulated mammalian TSC-22 sequence and is required during oogenesis. Mech Dev 65: 197–208. 9256356 10.1016/s0925-4773(97)00080-4

[6] Choi SJ, Moon JH, Ahn YW, Ahn JH, Kim DU, et al (2005) Tsc-22 enhances TGF-beta signaling by associating with Smad4 and induces erythroid cell differentiation. Mol Cell Biochem 271: 23–28. 15881652 10.1007/s11010-005-3456-7

[7] Yan X, Zhang J, Pan L, Wang P, Xue H, et al (2011) TSC-22 promotes transforming growth factor beta-mediated cardiac myofibroblast differentiation by antagonizing Smad7 activity. Mol Cell Biol 31: 3700–3709. 21791611 10.1128/MCB.05448-11 PMC3165719

[8] Kawamata H, Fujimori T, Imai Y (2004) TSC-22 (TGF-beta stimulated clone-22): a novel molecular target for differentiation-inducing therapy in salivary gland cancer. Curr Cancer Drug Targets 4: 521–529. 15379637 10.2174/1568009043332844

[9] Kawamata H, Nakashiro K, Uchida D, Hino S, Omotehara F, et al (1998) Induction of TSC-22 by treatment with a new anti-cancer drug, vesnarinone, in a human salivary gland cancer cell. Br J Cancer 77: 71–78. 9459148 10.1038/bjc.1998.11 PMC2151252

[10] Ohta S, Yanagihara K, Nagata K (1997) Mechanism of apoptotic cell death of human gastric carcinoma cells mediated by transforming growth factor beta. Biochem J 324 (Pt 3): 777–782. 10.1042/bj3240777 PMC1218492 9210400

[11] Iida M, Anna CH, Gaskin ND, Walker NJ, Devereux TR (2007) The putative tumor suppressor Tsc-22 is downregulated early in chemically induced hepatocarcinogenesis and may be a suppressor of Gadd45b. Toxicol Sci 99: 43–50. 17533171 10.1093/toxsci/kfm138

[12] Shostak KO, Dmitrenko VV, Vudmaska MI, Naidenov VG, Beletskii AV, et al (2005) Patterns of expression of TSC-22 protein in astrocytic gliomas. Exp Oncol 27: 314–318. 16404353

[13] Yu J, Ershler M, Yu L, Wei M, Hackanson B, et al (2009) TSC-22 contributes to hematopoietic precursor cell proliferation and repopulation and is epigenetically silenced in large granular lymphocyte leukemia. Blood 113: 5558–5567. 19329776 10.1182/blood-2009-02-205732 PMC2689053

[14] Nakamura M, Kitaura J, Enomoto Y, Lu Y, Nishimura K, et al (2011) TSC-22 is a negative-feedback regulator of Ras/Raf signaling: Implications for tumorigenesis. Cancer Sci. *online published*. 10.1111/j.1349-7006.2011.02108.x PMC11164176 21943131

[15] Lee JH, Rho SB, Park SY, Chun T (2008) Interaction between fortilin and transforming growth factor-beta stimulated clone-22 (TSC-22) prevents apoptosis via the destabilization of TSC-22. FEBS Lett 582: 1210–1218. 18325344 10.1016/j.febslet.2008.01.066

[16] Vogelstein B, Lane D, Levine AJ (2000) Surfing the p53 network. Nature 408: 307–310. 11099028 10.1038/35042675

[17] Yu J, Wang Z, Kinzler KW, Vogelstein B, Zhang L (2003) PUMA mediates the apoptotic response to p53 in colorectal cancer cells. Proc Natl Acad Sci U S A 100: 1931–1936. 12574499 10.1073/pnas.2627984100 PMC149936

[18] Yoon CH, Lee ES, Lim DS, Bae YS (2009) PKR, a p53 target gene, plays a crucial role in the tumor-suppressor function of p53. Proc Natl Acad Sci U S A 106: 7852–7857. 19416861 10.1073/pnas.0812148106 PMC2683089

[19] Kubbutat MH, Jones SN, Vousden KH (1997) Regulation of p53 stability by Mdm2. Nature 387: 299–303. 9153396 10.1038/387299a0

[20] Haupt Y, Maya R, Kazaz A, Oren M (1997) Mdm2 promotes the rapid degradation of p53. Nature 387: 296–299. 9153395 10.1038/387296a0

[21] Scheffner M, Huibregtse JM, Howley PM (1994) Identification of a human ubiquitin-conjugating enzyme that mediates the E6-AP-dependent ubiquitination of p53. Proc Natl Acad Sci U S A 91: 8797–8801. 8090726 10.1073/pnas.91.19.8797 PMC44693

[22] Scheffner M, Huibregtse JM, Vierstra RD, Howley PM (1993) The HPV-16 E6 and E6-AP complex functions as a ubiquitin-protein ligase in the ubiquitination of p53. Cell 75: 495–505. 8221889 10.1016/0092-8674(93)90384-3

[23] Nakashiro K, Kawamata H, Hino S, Uchida D, Miwa Y, et al (1998) Down-regulation of TSC-22 (transforming growth factor beta-stimulated clone 22) markedly enhances the growth of a human salivary gland cancer cell line in vitro and in vivo. Cancer Res 58: 549–555. 9458104

[24] Uchida D, Kawamata H, Omotehara F, Miwa Y, Hino S, et al (2000) Over-expression of TSC-22 (TGF-beta stimulated clone-22) markedly enhances 5-fluorouracil-induced apoptosis in a human salivary gland cancer cell line. Lab Invest 80: 955–963. 10879745 10.1038/labinvest.3780098

[25] Omotehara F, Uchida D, Hino S, Begum NM, Yoshida H, et al (2000) In vivo enhancement of chemosensitivity of human salivary gland cancer cells by over-expression of TGF-beta stimulated clone-22. Oncol Rep 7: 737–740. 10854535 10.3892/or.7.4.737

[26] Shostak KO, Dmitrenko VV, Garifulin OM, Rozumenko VD, Khomenko OV, et al (2003) Downregulation of putative tumor suppressor gene TSC-22 in human brain tumors. J Surg Oncol 82: 57–64. 12501169 10.1002/jso.10180

[27] Pomerantz J, Schreiber-Agus N, Liegeois NJ, Silverman A, Alland L, et al (1998) The Ink4a tumor suppressor gene product, p19Arf, interacts with MDM2 and neutralizes MDM2’s inhibition of p53. Cell 92: 713–723. 9529248 10.1016/s0092-8674(00)81400-2

[28] Zhang Y, Xiong Y, Yarbrough WG (1998) ARF promotes MDM2 degradation and stabilizes p53: ARF-INK4a locus deletion impairs both the Rb and p53 tumor suppression pathways. Cell 92: 725–734. 9529249 10.1016/s0092-8674(00)81401-4

[29] Sui G, Affar el B, Shi Y, Brignone C, Wall NR, et al (2004) Yin Yang 1 is a negative regulator of p53. Cell 117: 859–872. 15210108 10.1016/j.cell.2004.06.004

[30] Higashitsuji H, Itoh K, Sakurai T, Nagao T, Sumitomo Y, et al (2005) The oncoprotein gankyrin binds to MDM2/HDM2, enhancing ubiquitylation and degradation of p53. Cancer Cell 8: 75–87. 16023600 10.1016/j.ccr.2005.06.006

[31] Lohrum MA, Ludwig RL, Kubbutat MH, Hanlon M, Vousden KH (2003) Regulation of HDM2 activity by the ribosomal protein L11. Cancer Cell 3: 577–587. 12842086 10.1016/s1535-6108(03)00134-x

[32] Tang J, Qu LK, Zhang J, Wang W, Michaelson JS, et al (2006) Critical role for Daxx in regulating Mdm2. Nat Cell Biol 8: 855–862. 16845383 10.1038/ncb1442

[33] Colaluca IN, Tosoni D, Nuciforo P, Senic-Matuglia F, Galimberti V, et al (2008) NUMB controls p53 tumour suppressor activity. Nature 451: 76–80. 18172499 10.1038/nature06412

[34] Dai MS, Sun XX, Lu H (2008) Aberrant expression of nucleostemin activates p53 and induces cell cycle arrest via inhibition of MDM2. Mol Cell Biol 28: 4365–4376. 18426907 10.1128/MCB.01662-07 PMC2447154

[35] Lee ES, Yoon CH, Kim YS, Bae YS (2007) The double-strand RNA-dependent protein kinase PKR plays a significant role in a sustained ER stress-induced apoptosis. FEBS Lett 581: 4325–4332. 17716668 10.1016/j.febslet.2007.08.001

[36] Yoon CH, Miah MA, Kim KP, Bae YS New Cdc2 Tyr 4 phosphorylation by FdsRNA-activated protein kinase triggers Cdc2 polyubiquitination and G2 arrest under genotoxic stresses. EMBO Rep 11: 393–399. 20395957 10.1038/embor.2010.45 PMC2868534

[37] Lee SH, Son MJ, Oh SH, Rho SB, Park K, et al (2005) Thymosin {beta}(10) inhibits angiogenesis and tumor growth by interfering with Ras function. Cancer Res 65: 137–148. 15665289

