# Supplementary figures and images for: Crucial Role of TSC-22 in Preventing the Proteasomal Degradation of p53 in Cervical Cancer
Source: PLoS One. 2012 Aug 1;7(8):e42006. doi: 10.1371/journal.pone.0042006 (PMC3411576; doi:10.1371/journal.pone.0042006)

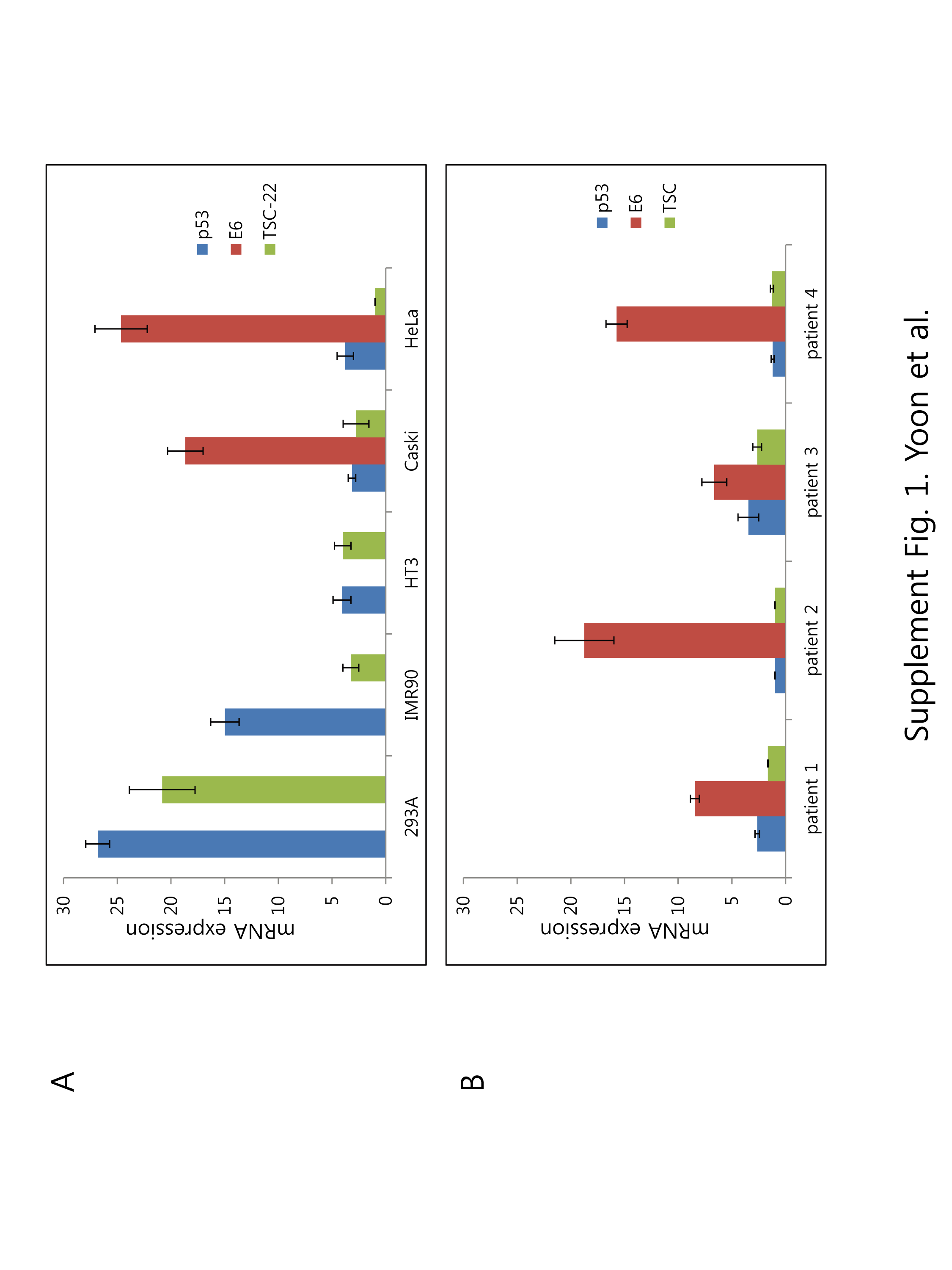

Supplement: Figure S1 — Quantification analysis of p53, E6 and TSC-22 genes. The mRNA expression of p53, E6 and TSC-22 in cervical cancer cell lines (A) and patients’ cancer specimens (B) was performed by quantitative RT- PCR by using GAPDH protein as reference gene. (TIF) [file pone.0042006.s001.tif]
